# Different approaches to HIV confirmation: a survey of three Italian regions

**DOI:** 10.3389/fcimb.2026.1775039

**Published:** 2026-07-06

**Authors:** Claudio Galli, Vincenza Regine, Anna Caraglia, Francesca Centrone, Maria Chironna, Gianluca Cruschelli, Massimo Farinella, Sara Moriconi, Valentina Annachiara Orlando, Chiara Pasqualini, Monia Puglia, Lucia Pugliese, Laura Rancilio, Lara Tavoschi, Fabio Voller, Barbara Suligoi

**Affiliations:** 1 Independent researcher, Roma, Italy; 2 National AIDS Unit, Department of Infectious Diseases, Italian National Institute of Health, Rome, Italy; 3 Former General Directorate of Health Prevention, Ministry of Health, Rome, Italy; 4 Hygiene Unit, Policlinico Hospital of Bari, Bari, Italy; 5 Hygiene Section, Interdisciplinary Department of Medicine, University of Bari, Bari, Italy; 6 Department of Translational Research and New Technologies in Medicine and Surgery, University of Pisa, Pisa, Italy; 7 Mario Mieli, LGBTQIA+ Culture Center, Rome, Italy; 8 Piedmont Regional Service for the Epidemiology of Infectious Diseases (SeREMI), Alessandria, Italy; 9 Research and Innovation Department (DAIRI), “SS Antonio e Biagio e C. Arrigo” University Hospital, Alessandria, Italy; 10 Tuscany Regional Health Agency (Agenzia Regionale di Sanità, ARS), Florence, Italy; 11 Caritas Ambrosiana, Milano, Italy

**Keywords:** diagnostic algorithms, HIV confirmation, HIV diagnosis, HIV-RNA, immunoblotting, predictive values

## Abstract

**Background:**

Accurate HIV diagnosis is critical to achieving global targets for ending the AIDS epidemic. Despite international recommendations, HIV confirmatory testing in Italy remains poorly standardized.

**Objectives:**

This study aimed to evaluate current HIV confirmation algorithms used in public laboratories across three Italian regions and suggest improvements for harmonization.

**Methods:**

Within the PRONTI project, we surveyed 31 second-level HIV laboratories in Piemonte, Toscana, and Puglia, representing 20% of the Italian population. Laboratory directors were interviewed using a structured questionnaire on first line and confirmatory assays, algorithm steps, and use of immunoblots (IB) and HIV-RNA testing.

**Results:**

All laboratories employed 4th-generation Ag/Ab immunoassays for initial screening. Four confirmatory algorithms were identified: (1) IB followed by HIV-RNA if IB negative/indeterminate (42%); (2) repeat 4th-generation assay, then IB and HIV-RNA if needed (36%); (3) direct HIV-RNA testing (16%); and (4) HIV-RNA followed by IB if RNA negative/inconclusive (6%). IB was used in all laboratories in different ways, and HIV-RNA testing in 87% of laboratories. Algorithms varied significantly by region, reflecting decentralized healthcare governance.

**Conclusions:**

HIV confirmation practices in Italy remain heterogeneous, with frequent reliance on IB despite recent international trends favoring molecular approaches. Algorithms prioritizing HIV-RNA testing offer faster results, reduce patient anxiety, limit HIV transmission and improve linkage to care, though IB remains useful in selected cases. National guidelines are urgently needed to standardize HIV diagnostic workflows and optimize resource use.

## 1 Introduction

Accurate HIV diagnosis is critical to achieving global targets for ending the acquired immune deficiency syndrome (AIDS) epidemic. Confirming positive results by HIV screening assays is a crucial step and several international organizations have issued recommendations on HIV confirmatory algorithms. However, HIV confirmatory testing in Italy remains poorly standardized.

**Figure 1:**
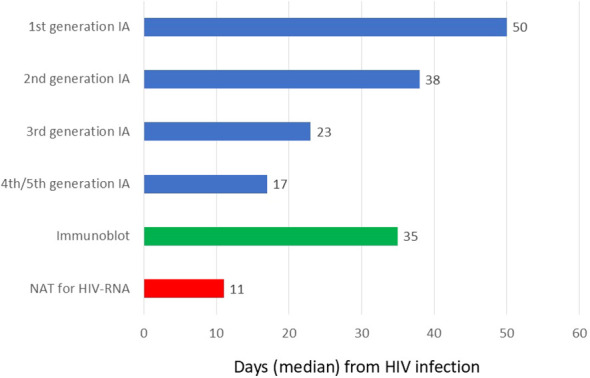
Estimated time of positivity, in days, from the acquisition of HIV infection with the different generations of HIV immunoassays, with currently employed HIV immunoblots and with nucleic acid amplification testing for HIV-RNA. The progressive narrowing of the initial diagnostic window by each generation of HIV immunoassays is linked to improvements in antigen purification and detection signal (2nd vs. 1st generation), to the adoption of a direct ‘sandwich’ format with the ensuing detection of IgM and IgG antibodies vs. IgG only (3rd generation vs. 2nd generation) and to the detection of the p24 antigen along with IgM/IgG antibodies (4th and 5th generation vs. 3rd generation). Testing for HIV-RNA by NAT further reduces the time to detection by 4–6 days compared to 4^th^/5^th^ generation immunoassays. Time is expressed as a median and calculated as an average from different sources (Fiebig et al, 2003; Branson, 2019). IA, immunoassays.

In 1983 a virus associated with the development of Acquired Immune Deficiency Syndrome (AIDS) was isolated by two different research groups in the US and France (Gallo et al, 1983; Barré-Sinoussi et al, 1983) and this started the quest for diagnostic tests that could detect the specific host immune response, with the dual purpose to identify the infected individuals and to prevent the spread of the infection. The initial tests were labeled by two different names (HTLV-III and LAV), according to the source (US or France, respectively) of the viral isolate employed for their development, but in 1986 that virus was officially named HIV (Human Immunodeficiency Virus) and all assays from then on were labeled as such. Over the years at least four generations of diagnostic tests have been developed: first generation assays employed viral proteins derived from virus cultures and could recognize only the IgG class antibodies, as well as the second generation assays which differed from the former ones due to the use of recombinant antigens and/or synthetic peptides representing the most immunogenic epitopes from the main viral antigens coded by the *env*, *pol* and *gag* genes of HIV, in addition to or instead of the viral lysate, and by further enhancements - the inclusion of specific antigens from HIV-2 and from the HIV-1 group O clade. A major breakthrough was reached in the early ‘90s of last century by the third-generation immunoassays, which adopted a direct, or ‘sandwich’, format in which recombinant antigens/synthetic peptides similar to those employed in 2^nd^ generation immunoassays are both on the solid phase and conjugated to the reaction revealing mixture. This format detected antibodies for all immunoglobulin subclasses, thus guaranteeing a better sensitivity by reducing the diagnostic ‘window’ period in the early phases of infection by 20 to 25 days compared to second generation assays (Branson, 2019). Lastly, fourth-generation enzyme and chemiluminescent immunoassays, which are called antigen/antibody (Ag/Ab) combination assays, have been developed (Eshleman et al, 2009). The change from the previous generation HIV assays is represented by the adoption of a solid phase, that is coated with antibodies to the HIV p24 antigen and with recombinant antigens of HIV-1 and HIV-2. The same components are present in the conjugate, allowing the simultaneous detection of both the HIV-1 p24 antigen and host antibodies. The p24 antigen appears in the blood before HIV antibodies are detectable, thus allowing an earlier detection of HIV infection by 4–8 days compared to 3^rd^ generation immunoassays (Fiebig et al, 2003; Eshleman et al, 2009). However, since the detection limit for the p24 antigen in acute HIV infections corresponds roughly to 30,000 copies of HIV-RNA/mL those HIV Ag/Ab combination assays provide a positive result on average five days after HIV-RNA detection (Fiebig et al, 2003; Gray et al, 2018; Branson, 2019) (**Figure 1**). A further refinement for HIV diagnosis has been the recent development of so called ‘fifth generation’ immunoassays (Salmona et al, 2014; Muhlbacher et al, 2019). Those assays have the same ‘sandwich’ format as the third and fourth-generation ones and their solid phase is coated either with monoclonal antibodies against HIV-1 p24 antigen or with several antigens from HIV-1 group M, HIV-1 group 2 and HIV 2.The addition of differently conjugated monoclonal anti-p24 and HIV-specific recombinant antigens/peptides allows the separate detection of HIV-1 p24 and of anti-HIV-1/HIV-2 antibodies. Nowadays, all virology and microbiology laboratories in Italy, as in most of the world, use 4th of 5th generation immunoassays as a first-level test, either for blood donation screening or for diagnosis, due to their higher sensitivity, and around 1 million tests are performed every year on outpatients (Galli et al, 2025). Rapid serological tests, either with 3rd or 4th generation technology, are also available but in Italy those tests are employed outside the hospital settings and no official data on the number of rapid tests performed are available. The sensitivity of 4^th^ and 5^th^ generation HIV tests is outstanding, and the specificity is also excellent, ranging between 99.7% and 99.9% (Alexander, 2016; Adhikari et al, 2018; Avidor et al, 2018; Parker et al, 2019; Muhlbacher et al, 2019; Huang et al, 2023); but due to the low prevalence of HIV infection in Italy, their positive predictive value (PPV) is relatively low. HIV testing therefore requires the adoption of adequate strategies to confirm positive first-level assays. Confirmatory assays for HIV antibody testing were developed alongside screening assays. The earliest tests relied on the Western blot technique, in which HIV antigens are separated by polyacrylamide gel electrophoresis and transferred to a membrane to detect specific antibodies to each viral antigen in a patient’s serum (CDC, 1989). Over time, Western blot was progressively replaced by other immunoblots (IBs), in which recombinant or synthetic peptides are individually “blotted” onto solid-phase strips for indirect immunoassays that identify antibody reactivity to different HIV−1 (and later also HIV−2) antigens (Thorpe et al., 1987; Pollet et al., 1991; Tobler et al., 1997; Branson, 2019. IBs have evolved substantially, and most now include four HIV−1 antigens (p24, p31, gp41, and gp160) and two HIV−2 antigens (gp36 and gp140) to differentiate HIV−1 from HIV−2 antibodies (Kondo et al., 2018). Although all IBs provide very high specificity—approaching 100%—their sensitivity is limited by the exclusive detection of IgG-class antibodies, the use of varying positivity criteria (AMCLI ETS, 2023) based on different band combinations, longer turnaround times, and reduced performance in acute or early infection compared with 4th- and 5th−generation HIV immunoassays (**Figure 1**).

The sensitivity gap of IB methods during the early phases of infection can be addressed by using nucleic acid amplification tests (NAT) for the qualitative and quantitative detection of HIV RNA, the earliest detectable marker of HIV infection (**Figure 1**). NAT has been incorporated into several confirmatory algorithms because HIV RNA is detectable throughout all stages of infection in individuals not receiving antiretroviral therapy (ART) (Branson, 2019).

Several recommendations on HIV confirmatory algorithms have been provided at international level, such as those issued in Spain (SEISDA, 2014), United States (CDC, 2018), United Kingdom (BHIVA, 2020, Germany (RKI) Robert Koch-Institut, 2022; France (HAS/CNS) Conseil national du sida et des hépatites virales, 2024; Switzerland (FOPH, Swiss Federal Office of Public Health, 2025) as well as at national level (AMCLI ETS, 2023) (**Table 1**) but the issue of HIV confirmation in Italy is still not standardized and unresolved. Therefore, as part of the ongoing PRONTI (Project on outpatient HIV testing in Italy) research project, financed by the Italian Ministry of Health (MoH), we aimed to determine which algorithms are currently used in a consistent number of Italian public laboratories, to compare the algorithms with those recommended by the guidelines issued in Western countries with similar dynamics of HIV infection and to identify which confirmatory algorithm shall be better suited to the Italian settings to make HIV testing and confirmation more standardized and efficient.

**Table 1:** Comparative overview of national HIV diagnostic algorithms in France, Germany, Italy, Switzerland, United Kingdom, USA.

Country and date of issue	Scientific society/regulatory body	Screening test (1st step)	Confirmatory test (2nd step)	Management of discordant/inconclusive results
Spain 2014	SEISIDA/GeSIDA (Sociedad Española Interdisciplinaria del Sida/Grupo de Estudio del Sida)	4th Gen Ag/Ab Combo	Immunoblot or rapid confirmatory test + 2^nd^ sample: 4th Gen Ag/Ab Combo	HIV-RNA (PCR)
USA 2018	Centers for Disease Control (CDC)	4th Gen Ag/Ab Combo	HIV-1/HIV-2 antibody differentiation immunoassay	HIV-1 RNA by NAT
United Kingdom 2020	BHIVA/BASHH (British HIV Association/British Association for Sexual Health and HIV)	4th Gen Ag/Ab Combo	Different Ag/Ab assay + HIV-1/2 Typing Assay	Immediate HIV-RNA (Viral Load) and repeat serology after 2 weeks
Germany 2022	DAIG/RKI (Deutsche AIDS-Gesellschaft/Robert Koch Institut)	4th Gen Ag/Ab Combo	Immunoblot (Western Blot or Line Immunoassay) or nucleic acid testing (NAT) for HIV-RNA	Repeat testing and Nucleic Acid Testing (NAT/PCR) If initial NAT is used for confirmation, repeat serology/immunoblot is recommended for legal/official validation.
Italy 2023	AMCLI (Italian Association of Clinical Microbiology)	4th Gen Ag/Ab Combo	HIV 1/2 immunoblot or different 4^th^ generation Ag/Ab assay + HIV-1/2 immunoblot	HIV-RNA (quantitative or qualitative)
France 2024	HAS/CNS (Haute Autorité de Santé/Conseil National du Sida)	4th Gen Ag/Ab Combo	In asymptomatic patients: Immunoblot + 2^nd^ sample: 4th Gen Ag/Ab Combo In symptomatic patients: quantitative HIV-RNA	Quantitative HIV-RNA
Switzerland 2025	FOPH (Federal Office of Public Health)	4th or 5th Gen Ag/Ab Combo	HIV-1/2 differentiation assay or HIV-RNA (quantitative or qualitative)	HIV-1/2 differentiation assay or HIV-RNA (quantitative or qualitative)

The recommendations are ordered from less to more recent. The table highlights the standardized use of 4^th^ generation screening assays and the specific confirmatory pathways recommended by regulatory bodies or scientific societies. In most instances serological testing, either by immunoblot or by a different 4^th^ generation HIV immunoassay and then immunoblot, is recommended as a second step while testing for HIV-RNA is used only on samples that are not confirmed at the 2^nd^ step. Testing for HIV-RNA as a second step is indicated in the guidelines from Germany, France (in symptomatic patients) and Switzerland. The link to access the different guidelines is reported in the References.

## 2 Materials and methods

The data presented in this study were searched in different official databases which we were authorized to access in the context of our research project. In detail, we searched the database on outpatients HIV testing, provided by the MoH, and the databases available from the Regional Health Agencies of three Italian regions involved in the PRONTI project, we identified the laboratories in charge of HIV confirmation diagnostic tests (called second-level HIV laboratories). Our search was limited to public laboratories included in the National Health System, since details on HIV testing in private laboratories are not available from the aforementioned databases nor from other reliable sources. Representatives of the Regional health Agencies from the three regions conducted a 1:1 interview with the laboratory directors of the selected sites proposing an ‘*ad hoc*’ survey that included questions on the type of first line and confirmatory HIV testing assays employed. Specifically, laboratory directors were asked: a) if they performed confirmatory HIV testing on samples coming from different public settings in their area of competence or if they reflected those samples to other laboratories; b) which diagnostic HIV assays were employed as a first test and for repeat testing, if needed; c) which algorithm was used to confirm HIV positivity by first line tests and/or or after repeat testing; d) if they processed samples coming from other sites; e) if they employed immunoblots (IB) as a first or second level test for HIV confirmation; f) which were the steps following a negative or indeterminate IB result; g) if the IB used was able to discriminate anti-HIV-1 and anti-HIV-2 antibodies; h) if testing for HIV-RNA by molecular amplification techniques was used at any step for HIV confirmation, and if yes at which step; i) if assays for the single determination of HIV-1 p24 antigen were used at any step during the confirmation process, and if yes at which step; j) if a second sample is required at any step during the confirmation process.

The questionnaires were then analyzed to ascertain which confirmation algorithm is mostly adopted at each site. The analysis has been performed for each of the three regions and in aggregate and data were analyzed by absolute numbers and percentages; for the latter, the significance between differences was calculated by chi square or Fisher’s exact test. Finally, the algorithms identified in this survey have been compared to the Italian recommendations and to the guidelines for HIV confirmation from six other Western countries with similar HIV epidemic pattern. The pros and cons of each one have been compared on the aim to propose the most appropriate algorithm for the current Italian public health and epidemiological situation.

## 3 Results

**Figure 2:**
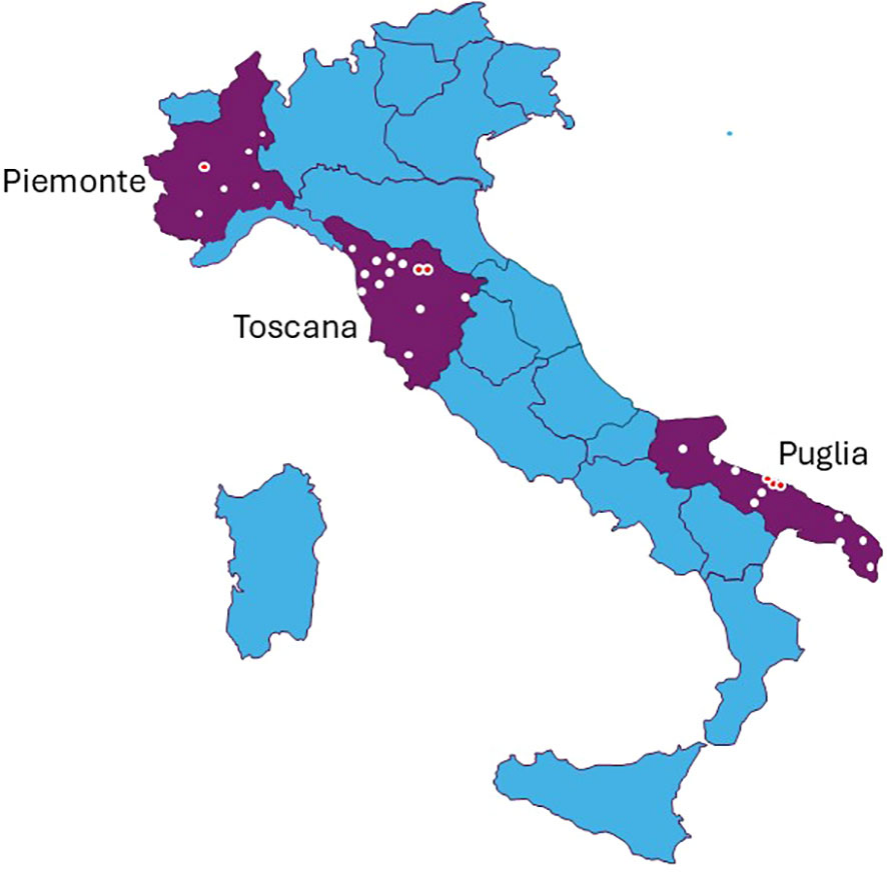
Distribution of participating laboratories in the three regions involved in the PRONTI project: Piemonte (North-West: inhabitants as of July 31st, 2025: 4,256,071), Toscana (Center: inhabitants as of July 31st, 2025: 3,661,386) and Puglia (South-East: inhabitants as of June 30th, 2025: 3,866,443). White dots indicate the location of second-level HIV laboratories that have been interviewed to obtain relevant information on HIV testing procedures and algorithms; red dots indicate laboratories that were interviewed and located in the region capital city.

The three regions involved in the project were Piemonte (North-West: inhabitants as of July 31st, 2025: 4,256,071), Toscana (Center: inhabitants as of July 31st, 2025: 3,661,386) and Puglia (South-East: inhabitants as of June 30th, 2025: 3,866,443). The total of 11,783,900 inhabitants in those regions represents 20% of the total Italian resident population. According to the MoH database, in the three regions 274,185 first-level assays and 1,402 IBs have been performed, corresponding to 25.0% of all first-level assays and 21.8% of all IBs performed in Italy. Thirty-one second level HIV testing laboratories were identified, distributed as follows: in Piemonte there were six laboratories located in the main city of five different provinces and in the region’s capital city, whereas in Puglia and Toscana the laboratories were more scattered and higher in number (thirteen in Toscana, twelve in Puglia) (**Figure 2**). All laboratories were located in public hospitals and six of them (19.4%) were in public University Hospitals. In all regions HIV testing was carried out in Clinical Microbiology/Virology or Clinical Pathology services except for one site in Puglia, where HIV confirmation was performed in a transfusion service as an additional task to their main activity which is the biological validation of blood donations for a large metropolitan area.

For first-line testing, 4th generation Ag/Ab combination immunoassays were used by all participating laboratories. A. All these assays are automated and guarantee a high level of sensitivity and specificity (Eshleman et al, 2009; Salmona et al, 2014; Alexander, 2016; White et al, 2018; Parker et al, 2019; Huang et al, 2023). A sample is considered positive when repeatedly reactive using the same assay. For confirmation, IBs by different manufacturers were used by all 31 laboratories, being a rapid immunochromatographic test with automated reading (Geenius HIV-1/2 Confirmatory Assay, Bio-Rad Laboratories, Hercules, California; USA) the most frequently employed. Two laboratories employed an anti-HIV-1-only IB whereas 29 (93.1%) laboratories used IBs that discriminate anti-HIV-1 from anti-HIV-2 antibodies. Twenty-seven laboratories also run a nucleic acid amplification test (NAT) for HIV-RNA. All HIV-RNA results were considered both qualitative and quantitative as they provide, respectively, evidence of the presence of HIV-RNA (when higher than detectability threshold of 50 RNA copies/ml) and the amount of RNA (when its concentration falls within the dynamic range of the assay). On the other hand, none of the 31 laboratories tested for HIV-1 p24 antigen alone.

**Figure 3:**
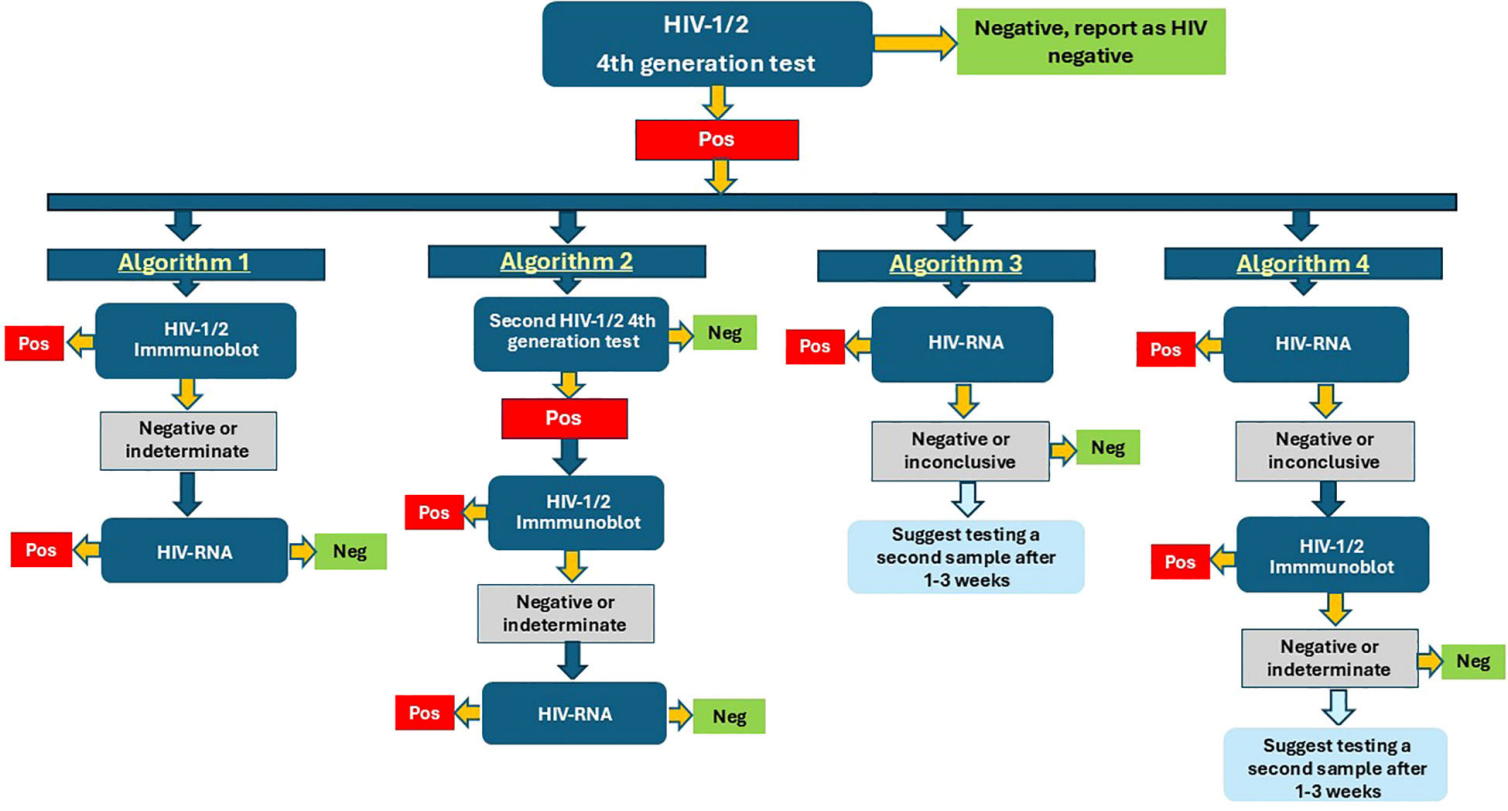
Flow charts of the four algorithms employed to confirm HIV positivity in 31 second level HIV laboratories in three Italian regions (in order of frequency of use). In all cases the algorithm starts with a first-level 4th generation HIV immunoassay and involves up to three subsequent steps to establish a result as HIV negative or positive. From left to right: Algorithm 1 - IB for HIV-1 and HIV-2 antibodies; if IB negative or indeterminate, HIV-RNA. Algorithm 2 – testing by another 4th generation Ag/Ab combination assay by a different manufacturer; if negative, result reported as negative; if positive, IB; if IB negative or indeterminate, HIV-RNA. Algorithm 3 - Samples are directly processed for HIV-RNA, with a negative or positive report according to that result. In case of inconclusive HIV-RNA result or high clinical suspicion of HIV infection a second serum sample may be requested after 1 to 3 weeks. Algorithm 4 - Samples are directly processed for HIV-RNA, with a negative or positive report according to that result. A negative or inconclusive result for HIV-RNA prompts testing by HIV 1/2 IB. This approach considers the possibility that the sample is obtained from an individual already HIV positive and on antiviral treatment with viral suppression. In case of a negative or indeterminate result by IB a second sample taken after 1–3 weeks may be requested. Neg, negative; Pos, positive.

**Figure 4:**
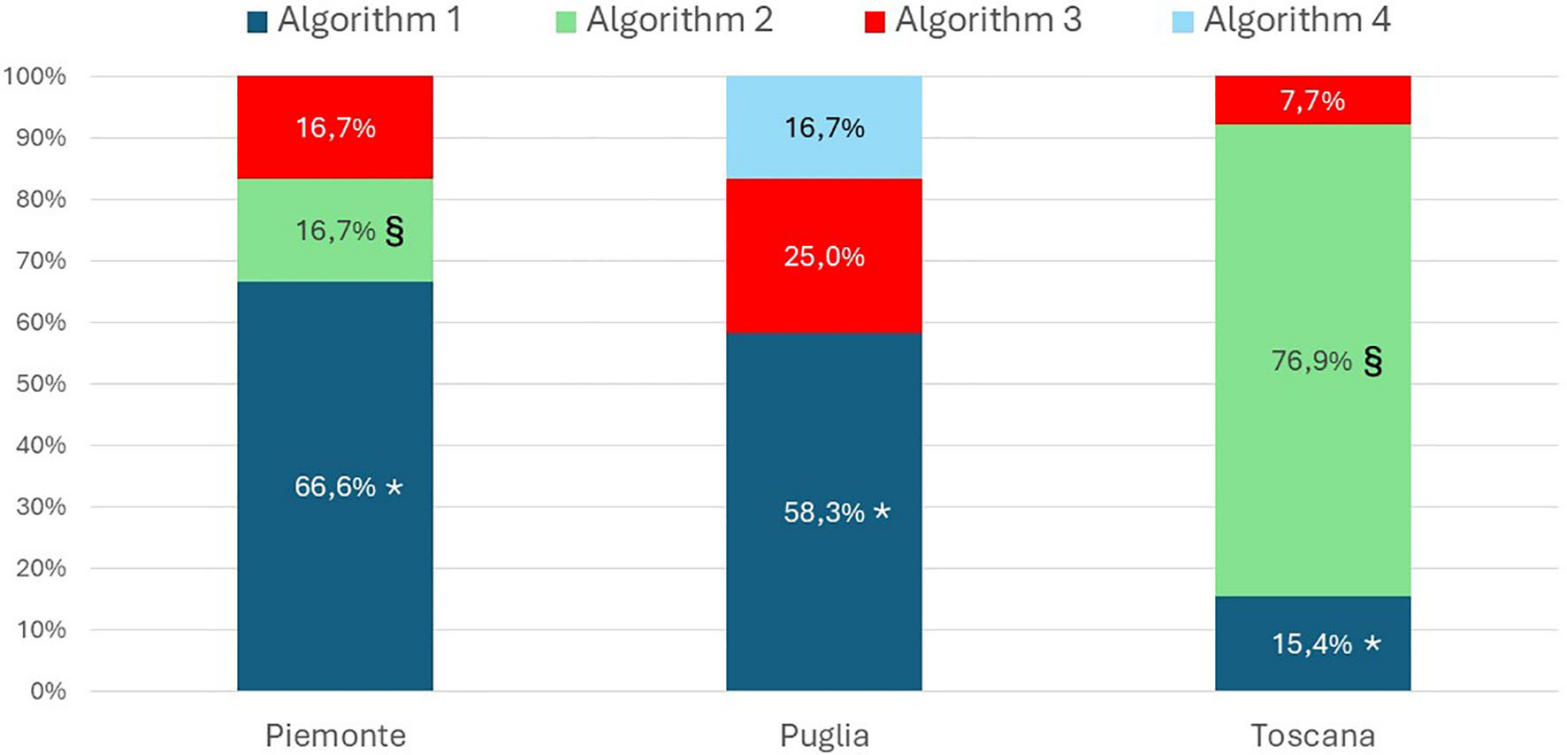
Percentage distribution of the four HIV confirmation algorithms in the three Italian Regions participating in the PRONTI project. Algorithm 1 was the most used among laboratories in Piemonte (66.6%) and Puglia (58.3%) and only by 15.4% of laboratories in Toscana (*p<0.01 by chi square) where most laboratories (76.9%) adopted Algorithm 2, which was used only in one laboratory in Piemonte (16.7%; §p<0.01) and by no laboratory in Puglia. Algorithm 3 is used in 16.7% of laboratories in Piemonte and 7.7% in Toscana whereas algorithms 3 and 4 were adopted by 41.7% of laboratories in Puglia. Overall, the algorithms suggested by the Association of Clinical Microbiology (that match Algorithm 1 and 2 in the present analysis), were employed in 24 out of 31 laboratories (77.4%).

While first line testing technology is consistent throughout the 31 laboratories, confirmatory algorithms following a positive first-level assay differed by region and within region. Four different confirmatory algorithms have been identified (**Table 2**; **Figures 3**, **4**). In detail and in order of frequency of use:

**Table 2:** HIV confirmatory algorithms employed by 31 reference laboratories in three Italian regions to confirm reactivity by a first-level HIV 1/2 4th generation diagnostic assay.

N.	N. of sites & frequency of use	Initial confirmatory test	Other confirmatory tests	Need for a 2nd sample	Time for final result	Cost ratio	Advantages	Disadvantages
1	13 42%	HIV 1/2 immunoblot	HIV-RNA, if IB negative or inconclusive	Infrequent	2–14 days	1	Quick differentiation between HIV-1 and HIV-2 antibodies; cost savings	Delayed turnaround time; disagreeing definitions of positivity based on different combinations of bands; delay in reporting results; increased anxiety for the individual
2	11 36%	Second 4^th^ generation HIV test	- HIV 1/2 IB if 2^nd^ immunoassay positive - HIV-RNA if IB negative or inconclusive	Frequent (reported by 54.5% of sites)	1–14 days	1,26	Quick reporting of negative results	Reduced sensitivity in acute or early infections if second 4^th^ generation test is less sensitive; delayed turnaround time; frequent need of a 2^nd^ sample; cost increase compared to algorithm 1; delay in reporting results; increased anxiety for the individual
3	5 16%	HIV-RNA	None	Rare	1–5 days	1,65	Faster results; lower workload for the laboratory; reduced anxiety for the individual; timely linkage to care and treatment initiation	Risk of false-negative results in 2-4% of cases on ART or PrEP with undetectable RNA; cost increase compared to algorithm 1
4	2 6%	HIV-RNA	IB if HIV-RNA negative or inconclusive	Rare	1–7 days	1,68	Faster results; lower workload for the laboratory; reduced anxiety for the individual; timely linkage to care and treatment initiation	Delayed turnaround time when HIV-1/2 IB is needed; cost increase compared to algorithm 1

The four algorithms are reported in order of frequency of use. The time to final result has been reported as a range based on the answers collected from the 31 reference sites; longer times are linked to the use of HIV immunoblots and/or to the need to obtain a second sample. The cost ratio of each confirmation algorithm compared to algorithm 1 has been estimated considering a 0.3% HIV prevalence in Itay (Regine et al., 2025), a 99.8% specificity of the 4th generation HIV 1/2 immunoassays (Adhikari et al., 2018; Avidor et al, 2018; Muhlbacher et al, 2019; Huang et al, 2023) and the reimbursement according to Italian MoH. HIV 1/2: human immunodeficiency viruses 1 and 2; IB= immunoblots for HIV-1/2 antibodies; HIV-RNA= nucleic acid amplification methods for HIV-RNA.

3.1 Algorithm 1 (13 sites, 42%) envisions the use of an IB for HIV-1 and HIV-2 antibodies (HIV-1/2 IB). Samples positive by IB are reported as HIV positive while testing for HIV-RNA is performed if IB is negative or indeterminate.
3.2 Algorithm 2 (11 sites, 36%) is similar to algorithm 1 but in case of a positive result by the first-line assay requires retesting by a 4th generation Ag/Ab combination assay from a different manufacturer. Negative results on the second assay are ruled as false positive at initial testing, and the sample is reported as negative. Samples that are positive by the second Ag/Ab combination assay are processed by IB, and then by HIV-RNA if IB is negative or indeterminate. Of note, 6 out of 11 sites (54.5%) adopting this algorithm require recalling the person after the first test to have blood drawn for a second sample.
3.3 Algorithm 3 (5 sites, 16%): samples are processed for HIV-RNA and reported as negative or positive according to the ensuing result. However, in some instances (evidence of HIV acute infection, person known to undergo ART or on pre-exposure prophylaxis), a negative or inconclusive RNA result may require a second sample collected after 1 to 3 weeks.
3.4 Algorithm 4 (2 sites, 6%): samples are tested for HIV-RNA and a negative or inconclusive result prompts testing by HIV-1/2 IB. This approach considers the possibility that the sample has been obtained from an HIV positive individual under ART with viral suppression who did not report his/her serostatus and treatment or was not able to convey the information.

The cost of each confirmatory strategy can be estimated by assuming the expected frequency of positive results on first−line assays, based on the estimated 0.3% HIV prevalence in Italy (Regine et al., 2025) and the average 99.8% specificity of 4th− and 5th−generation HIV immunoassays (Alexander, 2016; Adhikari et al., 2018; Avidor et al, 2018; Parker et al., 2019; Muhlbacher et al., 2019; Huang et al., 2023). Testing 10,000 samples would yield approximately 50 positive results at initial screening. According to current reimbursement rules in Italy, algorithm 1 is the most cost−effective option, whereas all other algorithms result in higher expenditures (+26% for algorithm 2, + 65% for algorithm 3, and +66% for algorithm 4).

The frequency of use of every algorithm varied across the three participating regions (**Figure 4**). Algorithm 1 was the most used among laboratories in Piemonte (66.6%) and Puglia (58.3%) and only by 15.4% of laboratories in Toscana (p<0.01 by chi square vs. Piemonte and Puglia) where most laboratories (76.9%) adopted Algorithm 2, which was used only in one laboratory in Piemonte (16.7%; p<0.01 by Fisher’s exact test) and by no laboratory in Puglia. Algorithm 3 is used in 16.7% of laboratories in Piemonte and 7.7% in Toscana whereas algorithms 3 and 4 were adopted by 41.7% of laboratories in Puglia. Overall, the algorithms suggested by the Association of Clinical Microbiology (that match Algorithm 1 and 2 in the present analysis), were employed in 24 out of 31 laboratories (77.4%).

## 4 Discussion

The Joint United Nations Programme on HIV/AIDS (UNAIDS) made a global commitment to end the Acquired Immune Deficiency Syndrome (AIDS) epidemic as a public health threat by 2030 and redefined a continuum of care to achieve that goal by targeting 95% of infections being diagnosed, 95% of those diagnosed being treated, and 95% of those treated achieving viral suppression (UNAIDS, 2023). Of course, the first step is of utmost relevance to develop the whole continuum of care, and efforts have been made over the last years to reduce the proportion of undiagnosed individuals by promoting HIV testing, facilitating access to and trying to reduce barriers by incorporating HIV testing in routine care, promoting opt-out testing and reducing stigma and discrimination. Despite all these efforts, according to European Centers for Disease Prevention and Control (ECDC) about 8% of the estimated 625,000 people living with HIV in the European Union and European Economic Area (EU/EEA) are still undiagnosed (ECDC, 2025), and a slightly lower percentage of undiagnosed HIV cases is estimated also in Italy (Regine et al, 2025; Navarra et al, 2025).

The three pillars of HIV diagnosis are rapidity, accuracy and sustainability (World Health Organization, 2021; Patel et al, 2025). The acceptance threshold, that may be defined as a ‘confidence interval’, for each of the three shall vary according to the setting and the environment. As an example, an approach mainly based on hospital testing by assays working on automated laboratory platforms may be sustainable and reasonably fast in high resource countries but not in low resource settings, where rapid tests or point-of-care assays may be preferred. Testing accuracy of most currently available first-line assays guarantees very high levels both in sensitivity and specificity (Branson, 2019). When testing by 4th generation Ag/Ab combination assays, likelihood of detecting HIV infection both in routine screening and individuals at high risk is almost 100% in established infections and very high also in the initial stages of infection, showing the high sensitivity of the assays. These assays detect HIV in over 80% of antibody-negative individuals that could be diagnosed only by HIV-RNA testing (Eshleman et al, 2009; White et al, 2018; Baltaro et al., 2019). On the other hand, these assays guarantee also excellent specificity, ranging from 99.7% to 99.9% according to published evidence (Alexander, 2016; Adhikari et al, 2018; Avidor et al, 2018; Parker et al, 2019; Muhlbacher et al, 2019; Huang et al, 2023), and this breeds confidence in a reactive result being indeed positive in most circumstances. Nevertheless, the individual and social burden of a false positive result is high and even if this may occur only in 1–2 cases over 1000 tests performed the risk is too high to ignore the need for subsequent testing to confirm HIV positivity. On this purpose, for decades a standard approach has been followed by testing samples reactive to any first-level assay by IBs, but different algorithms have been proposed.

From data collected in our survey, HIV confirmation in Italy is not standardized and still far from being harmonized. While a diagnostic workflow for HIV infection has been proposed and recently updated by the Italian Society for Clinical Microbiology (AMCLI ETS, 2023), there are no official guidelines. The ‘AIDS Law’ issued in June 1990 (Italian Government, 1990) does not include any national recommendation on laboratory HIV testing procedures and no other official committee has undertaken this task. Considering that in Italy health care is administered individually by the 21 regions/provinces, it is not surprising to observe procedural differences, in some cases quite substantial, on HIV diagnostic laboratory practices (**Figure 4**).

A first result that stems from our analysis is the uneven distribution of HIV confirmation laboratories: one region (Piemonte, the largest one in terms of inhabitants) has few sites located in provinces, whereas Puglia and Toscana besides provincial laboratories have additional sites, generally distributed according to population density: in both these regions, the regional capital has 2–3 such laboratories.

From our survey, a high degree of homogeneity appears in the method used for HIV positivity confirmation, IBs (or analogue methods) being employed across the 31 laboratories, possibly reflecting the current offer from the diagnostic industry. On the other hand, the combination and sequence of methods included in an algorithm for HIV diagnosis shows differences across and within regions. Our project includes three out of 21 regions representing approximately 20% of the total Italian population and 25% of all HIV tests performed in outpatients. The Italian health care system is ruled by regional administration that impacts on hospital and laboratory organization and expenditures. Therefore, laboratories can decide at regional level (sometimes even at provincial level) different approaches for diagnostic protocols based on local needs. These differences emerge in our analysis showing quite a different distribution of the four testing algorithms in the three regions. With the aim of integrating the information obtained on algorithms used in the three above-mentioned regions, we conducted a personal interview with representatives of major HIV reference laboratories located in other three regions (North: Emilia-Romagna; Center: Lazio; South: Campania). These laboratories reported using in most instances the confirmation algorithm that we defined as algorithm 1 (IB followed by HIV-RNA when necessary) while testing directly for HIV-RNA in selected cases.

At the global level - and particularly in settings with high HIV prevalence (5% or higher) - the World Health Organization (WHO) no longer recommends the use of immunoblots (IB) because of their high cost and long turnaround time. WHO instead recommends HIV testing algorithms that achieve a positive predictive value of at least 99% and that combine assays with ≥99% sensitivity and ≥98% specificity. Depending on the HIV prevalence in the population being tested, this approach requires two or three sequential reactive tests (WHO, 2021). While this strategy is appropriate for low−resource or high−burden settings, the approach to HIV confirmation differs substantially in high−income countries. We compared current recommendations for HIV diagnosis in Italy with those adopted in six other Western countries that have an epidemiological profile similar to Italy (**Table 1**). In all six countries, the first step consists of a 4th− or 5th−generation screening assay. The algorithm currently recommended by the U.S. Centers for Disease Control and Prevention (CDC), as well as by Spain and Germany, requires that reactive samples undergo HIV−1/HIV−2 antibody IB testing. If the IB result is negative or indeterminate, HIV RNA testing is performed to confirm HIV infection or to classify the initial reactivity as a false−positive result (CDC, 1989).

This is also the current recommendation in Italy, as outlined in the “HIV diagnostic pathway” issued by the National Association of Clinical Microbiology (AMCLI ETS, 2023). However, these recommendations also allow for an alternative approach: performing a second 4th−generation immunoassay on samples reactive on the initial screening test, proceeding to IB only if the second assay is positive, and then testing for HIV RNA in samples not confirmed by IB. This strategy is also used in the United Kingdom but not in the other five countries examined. Conversely, using HIV RNA as the first confirmatory step is recommended only in France - for symptomatic individuals - and in Switzerland, where laboratories may choose either to perform IB followed by HIV RNA for inconclusive cases or to reverse the order, mirroring algorithms 1 and 4 identified in our study.

In our view, algorithms that rely on immunoblots (IBs) as the first step in HIV confirmation - still recommended in Italy, endorsed by most international guidelines, and used in the majority of centers we surveyed - are no longer appropriate. As previously noted, WHO does not recommend IBs in the diagnostic workup for HIV infection because of their high cost in low−resource settings, reduced sensitivity during acute or very recent infection, long turnaround times, and inconsistent positivity criteria based on different combinations of antibody bands. While cost may be a less relevant factor in high−income countries, the remaining limitations are objective and interconnected: the reduced sensitivity of IBs in early infection necessitates HIV RNA testing, which in some cases requires a second specimen and further prolongs the time to diagnosis. Delayed reporting has negative consequences for patients, increasing anxiety and potentially contributing to onward transmission, which is highest during the early stages of infection (Kaperak et al., 2023). Moreover, different guidelines propose different combinations of antibody bands to define a positive HIV result (AMCLI ETS, 2023), and the frequency of indeterminate results - which require additional testing - varies depending on the criteria adopted (AMCLI ETS, 2023) and on the specific IB used (Kondo et al., 2018). The option we identified as algorithm 2 (a second 4th−generation immunoassay followed by IB and HIV RNA testing) has been proposed to reduce the number of IBs by limiting false−positive results at initial screening. Although this approach may offer some economic advantages - given that screening assays are generally less expensive than IBs – in Italy the cost will be actually higher compared to algorithm 1. Also, algorithm 2 carries the same drawbacks as algorithm 1, with the added disadvantage of an additional procedural step that increases the risk of laboratory error (Plebani, 2006) and further delays reporting, often compounded by the frequent need for a second specimen, with a high probability of ‘losing’ the patient, and increased levels of anxiety for the individual. Last, but absolutely not least, if the second HIV 4th generation assay is less sensitive than the first-level assay there is a risk of ‘missing’ positive samples collected during acute HIV infection.

The drawbacks of IB-based confirmatory algorithms can be overcome by a simpler algorithm that, after an initial reactive result, skips any second or third HIV immunoassay and IB, and processes samples straight for HIV-RNA (algorithm 3 in our survey) (Patel et al, 2010). This algorithm is considered preferable compared to the previous ones for several reasons. It is easier to perform because it entails only two testing steps, thus limiting the inherent risk of errors when more diagnostic steps are needed, it is faster in providing the result, it provides information on individual’s HIV antibody and virological level, it is more respectful of the patient by reducing apprehension related to longer waiting time to test results, and favors a more rapid linkage to care and treatment (Kaperak et al, 2023). A shortcoming of this algorithm is that an undetectable HIV-RNA has been reported in 2-4% samples reactive by 4th generation immunoassays and confirmed as positive by IB (Owen et al, 2008; Patel et al, 2010). This false negative result has been most frequently observed in samples from people on ART and in viral suppression (Avelino-Silva et al., 2026), when this information was not known or not reported at the time of HIV testing.

The diagnosis of HIV infection in individuals using HIV pre−exposure prophylaxis (PrEP) can also be challenging (Delaugerre et al., 2017), although HIV seroconversion among PrEP users is uncommon and was recently reported at 0.9% (Martin et al., 2025). Case reports describing breakthrough HIV infections in PrEP users have documented low and inconsistent reactivity on both HIV RNA assays and 4th−generation serologic tests (Delaugerre et al., 2017; Zucker et al., 2018; Avelino-Silva et al., 2024). As early as 2021, the Centers for Disease Control and Prevention recommended the combined use of laboratory−based Ag/Ab assays and HIV RNA testing for individuals who are currently taking or have recently taken PrEP or HIV post−exposure prophylaxis (PEP) (CDC, 2021), underscoring the advantages of the algorithm we identified as no. 3.

Limitations in HIV detectability among individuals with prior exposure to antiretrovirals are further reflected in a recent publication by the U.S. Food and Drug Administration, which recommends a three−month deferral from the last dose of oral PrEP and a two−year deferral from the last dose of injectable PrEP for prospective blood donors (US FDA, 2023). In the rare instances in which HIV RNA tests negative despite strong reactivity on first−line immunoassays, a supplemental HIV−1/2 IB should be performed (Branson, 2019). A key retrospective study (Pitasi et al., 2020) demonstrated that this algorithm correctly classified all specimens from early infection, all false−reactive screening specimens, and most specimens from established infection.

The choice of an HIV diagnostic algorithm has implications across several domains. A summary of the advantages and disadvantages of the four HIV confirmation algorithms used in the 31 participating laboratories is presented in **Table 2**. From a laboratory perspective, all options provide specificity and positive predictive value (PPV) approaching 100%. In all cases, sensitivity is not determined by the confirmatory algorithm itself (except for algorithm 2, if the second immunoassay is less sensitive than the first−line assay), but rather by the inherent sensitivity gap of all 4th− and 5th−generation HIV immunoassays during the early stages of infection (**Figure 1**) (Fiebig et al., 2003; Branson, 2019). The negative predictive value (NPV) of HIV testing therefore depends on the relative frequency of early infections: if early infections account for approximately 6% of all HIV diagnoses, as reported in a comprehensive study (Avidor et al., 2018), the NPV would exceed 99.9%. From an economic standpoint, under current reimbursement policies in Italy, algorithms 2, 3, and 4 are more costly than algorithm 1. However, the difference has limited impact on the overall cost of HIV testing because of the low proportion of samples that test positive at initial screening. From a clinical standpoint, algorithms that prioritize HIV RNA testing ensure faster reporting of positive results, reducing patient anxiety, facilitating prompt linkage to care and treatment initiation, and decreasing the potential for onward transmission.

Considering all these factors—and given that almost all second−level laboratories responsible for confirmatory HIV testing in Italy are equipped to perform HIV RNA assays—we believe that, from both a diagnostic and clinical perspective, it is advisable to adopt a confirmation strategy that includes HIV RNA testing, preferably in a reflex format using the same specimen, for samples that test positive with a 4th− or 5th−generation immunoassay. Notably, the international recommendations that move in this direction (France and Switzerland) are also the most recent (2024 and 2025). Algorithm 3, which calls for direct testing for HIV-RNA after a reactive HIV 4th generation test, appears efficient in terms of accuracy and time to result. Kaperak et al (Kaperak et al, 2023). demonstrate that this algorithm decreased significantly the number of discordant results compared to IB. An improvement in the identification of acute HIV infections, both in number and in time to diagnosis, was also observed, as the confirmation algorithm was efficient in differentiating acute infections (where IB can be negative due to the absence of antibodies) from false positives. Algorithm 4 (HIV-RNA and IB on negative/inconclusive samples) has been applied in a very recent study carried out in the US (Pinar Bilir et al, 2025). The Authors simulated HIV testing in a population of 24,400 adults, 0.72% of whom would have tested positive by a 4th generation HIV assay according to historical records. When the standard CDC algorithm (Centers for Disease Control, 2019) was employed, 8.78% of the samples would have needed HIV-RNA testing because of a negative or indeterminate IB result, whereas if HIV-RNA had been employed first only 1.95% of samples would have needed retesting by IB. By using Algorithm 4, the total number of confirmatory tests would decrease by 21.3% and retests would have been 4.5% instead of 20.7% when the CDC algorithm is employed. Applying Algorithm 4 in Italy would decrease the number of confirmatory tests compared to algorithms that are more frequently used. Moreover, it would be more efficient in the identification of acute and early-stage infections and reduce individual apprehension and concern in that time to result is shorter, allowing also for a faster linkage to care and prompt ART initiation in case of HIV positivity.

We therefore recommend option 4 (HIV RNA testing while retaining IB as a supplemental assay in selected cases) as the approach best suited to the current Italian context. We also advocate for the urgent development and implementation of national guidelines to harmonize HIV confirmatory testing across all regions, led by a multidisciplinary committee including experts in public health, virology, epidemiology, infectious diseases, and representatives from affected communities.

The current priorities of the Italian Ministry of Health in the HIV sector include improving access to HIV testing, implementing prevention strategies through information and education, optimizing linkage to and retention in care, and reducing the number of undiagnosed cases. In addition, systematic monitoring and evaluation of diagnostic outcomes, laboratory data reporting, and patient linkage to care will be essential to assess the real−world impact of the chosen algorithm.

## 5 Conclusions

The large differences noticed in Italy in algorithms used for laboratory HIV diagnosis, the absence of standardized national protocols, and the impact of various laboratory procedures at several levels, highlight the need for an agreed consensus on HIV laboratory diagnostic protocols.

A more efficient use of the technology that is currently available in all second-level laboratories in Italy suggests testing for HIV-RNA on samples reactive by 4th generation Ag/Ab HIV immunoassays followed by IB in case of a negative or inconclusive result for HIV-RNA. This approach provides an HIV positive report ready within a few days or even on the same day. The increase in cost compared to current practice is largely offset by the benefit of a rapid result, a reduced worry and concern for the individual waiting for the report, and a prompt linkage to care and ART initiation for true positives.

The relevant disparities observed among and within regions indicate the importance of establishing an expert committee including public health, virology, epidemiology, and infectious disease specialists as well as representatives of patients’ and non-governmental organizations active in the HIV/AIDS sector, with the purpose of elaborating national guidelines on the still unresolved issues of HIV laboratory diagnostic procedures.

## Data Availability

The data analyzed in this study is subject to the following licenses/restrictions: The dataset employed for this study is not publicly available due to constraints from the public institutions that provided the data. Requests to access these datasets should be directed to not applicable, see above.

## References

[B1] Adhikari E. H. Macias D. Gaffney D. White S. Rogers V. L. McIntire D. D. . (2018). Diagnostic accuracy of fourth-generation ARCHITECT HIV Ag/Ab Combo assay and utility of signal-to-cutoff ratio to predict false-positive HIV tests in pregnancy. Am. J. Obstet. Gynecol. 19, 408.e1–408.e9. doi:10.1016/j.ajog.2018.06.008 29913173

[B2] Alexander T. S. (2016). Human immunodeficiency virus diagnostic testing: 30 years of evolution. Clin. Vaccine Immunol. 23, 249–253. doi:10.1128/CVI.00053-16 26936099 PMC4820517

[B3] AMCLI ETS (2023). “ Percorso diagnostico “Infezioni da HIV”–Rif. 2023-03, rev. 2023,” in Italian association for clinical microbiology. Available online at: https://www.amcli.it/wp-content/uploads/2024/02/2023-03_INFEZIONI-DA-HIV.pdf (Accessed November 26, 2025).

[B4] Avelino-Silva V. I. Stone M. Bakkour S. Di Germanio C. Schmidt M. Conway A. L. . (2024). Suppressed HIV antibody responses following exposure to antiretrovirals-evidence from PrEP randomized trials and early antiretroviral treatment initiation studies. Int. J. Infect. Dis. 148, 107222. doi:10.1016/j.ijid.2024.107222 39186969 PMC11569788

[B5] Avelino-Silva V. I. Stone M. Di Germanio C. Lanteri M. C. Bakkour S. Grebe E. . (2026). HIV serologic reactivity varies with time of ART initiation in persons on long-term ART. J. Clin. Microbiol. 64, e0127325. doi:10.1128/jcm.01273-25 41406029 PMC12802137

[B6] Avidor B. Chemtob D. Turner D. Zeldis I. Girshengorn S. Matus N. . (2018). Evaluation of the virtues and pitfalls in an HIV screening algorithm based on two fourth generation assays - A step towards an improved national algorithm. J. Clin. Virol. 106, 18–22. doi:10.1016/j.jcv.2018.06.017 30007138

[B7] Baltaro R. J. Malenie R. Melbourne H. Garcia F. Gould E. W. Renshaw A. A. (2019). Risk stratification of HIV infection for patients needing molecular confirmation with the Abbott 4th generation Architect System. J. Clin. Virol. 113, 31–34. doi:10.1016/j.jcv.2019.02.004 30844622

[B8] Barré-Sinoussi F. Chermann J. C. Rey F. Nugeyre M. T. Chamaret S. Gruest J. . (1983). Isolation of a T-lymphotropic retrovirus from a patient at risk for acquired immune deficiency syndrome (AIDS). Science 220, 868–871. doi:10.1126/science.6189183 6189183

[B9] Branson B. M. (2019). HIV diagnostics. Current recommendations and opportunities for improvement. Infect. Dis. Clin. North. Am. 33, 611–628. doi:10.1016/j.idc.2019.04.001 31239094

[B10] British HIV Association British Association for Sexual Health and HIV British Infection Association (2020). “ Adult HIV testing guidelines 2020,” in BHIVA(London). Available online at: https://bhiva.org/clinical-guideline/hiv-testing-guidelines/ (Accessed November 26, 2025).

[B11] Centers for Disease Control (CDC) (1989). Interpretation and use of the Western blot assay for serodiagnosis of human immunodeficiency virus type 1 infections. MMWR Morb. Mortal. Wkly. Rep. 38, 1–7. doi:10.1037/e364152004-001 2501638

[B12] Centers for Disease Control and Prevention (2021). Preexposure prophylaxis for the prevention of HIV infection in the United States—2021 update clinical practice guideline. Available online at: https://www.cdc.gov/hiv/pdf/risk/prep/cdc-hiv-prepguidelines-2021.pdf (Accessed November 26, 2025).

[B13] Centers for Disease Control and Prevention Association of Public Health Laboratories (2018). Quick reference guide: Recommended laboratory HIV testing algorithm for serum or plasma specimens. Available online at: https://stacks.cdc.gov/view/cdc/50872 (Accessed November 26, 2025).

[B14] Conseil national du sida et des hépatites virales (2024). Prévention et dépistage de l’infection VIH. Available online at: https://cns.sante.fr/sites/cns-sante/files/2024/11/VIH-Prevention-et-depistage_Argumentaire_Rapport-dexperts_241118_ER.pdf (Accessed November 26, 2025).

[B15] Delaugerre C. Antoni G. Mahjoub N. Pialoux G. Cua E. Pasquet A. . (2017). Assessment of HIV screening tests for use in preexposure prophylaxis programs. J. Inf. Dis. 216, 382–386. doi:10.1093/infdis/jix297 28666370

[B16] Eshleman S. H. Khaki L. Laeyendecker O. Piwowar-Manning E. Johnson-Lewis L. T. Husnik M. . (2009). Detection of individuals with acute HIV-1 infection using the ARCHITECT® HIV Ag/Ab Combo assay. J. Acquir. Immune Defic. Syndr. 52, 121–124. doi:10.1097/QAI.0b013e3181ab61e1 19506484 PMC2744045

[B17] European Centers for Disease Prevention and Control (ECDC) (2025). HIV/AIDS Surveillance in Europe 2025–2024 data. Available online at: https://www.ecdc.europa.eu/sites/default/files/documents/2025-Annual-HIV-Report.pdf (Accessed November 26, 2025).

[B18] Fiebig E. W. Wright D. J. Rawald B. D. Garrett P. E. Schumacher R. T. Peddada L. . (2003). Dynamics of HIV viremia and antibody seroconversion in plasma donors: Implications for diagnosis and staging of primary HIV infection. AIDS 17, 1871–1879. doi:10.1097/00002030-200309050-00005 12960819

[B19] Galli C. Regine V. Caraglia A. Centrone F. Chironna M. Cruschelli G. . (2025). Outpatient testing for HIV in Italy 2018–2023 - Preliminary data. Microorganisms 13, 655. doi:10.3390/microorganisms13030655 40142547 PMC11946390

[B20] Gallo R. C. Sarin P. S. Gelmann E. P. Robert-Guroff M. Richardson E. Kalyanaraman V. S. . (1983). Isolation of human T-cell leukemia virus in acquired immune deficiency syndrome (AIDS). Science 220, 865–867. doi:10.1126/science.6601823 6601823

[B21] Gray E. R. Bain R. Varsaneux O. Peeling R. W. Stevens M. M. McKendry R. A. (2018). p24 revisited: A landscape review of antigen detection for early HIV diagnosis. AIDS 32, 2089–2102. doi:10.1097/QAD.0000000000001982 30102659 PMC6139023

[B22] Huang Y. Liu H. Dai S. Lan X. Liu S. Ren X. . (2023). Evaluation of a two-test algorithm for HIV screening in a low-prevalence setting and the indications for optimizing clinical management. Heliyon 9, e19400. doi:10.1016/j.heliyon.2023.e19400 37681153 PMC10481286

[B23] Italian Government . (1990). “ Law 135, 5th june, (1990). Programma di (Rome, Italy) interventi urgenti per la prevenzione e la lotta contro l'AIDS,” in Gazz. Uff. 132, 5–11

[B24] Kaperak C. Eller D. Devlin S. A. Hall A. Schmitt J. Friedman E. E. . (2023). Reflex human immunodeficiency virus (HIV) type 1 RNA testing enables timely differentiation of false-positive results from acute HIV infection. Open Forum Infect. Dis. 12, ofad629. doi:10.1093/ofid/ofad629 38269050 PMC10807991

[B25] Kondo M. Sudo K. Sano T. Kawahata T. Itoda I. Iwamuro S. . (2018). Comparative evaluation of the Geenius HIV 1/2 confirmatory assay and the HIV-1 and HIV-2 Western blots in the Japanese population. PloS One 13, e0198924. doi:10.1371/journal.pone.0198924 30379808 PMC6209130

[B26] Martin C. E. Ramatsoma H. Chidumwa1 G. Cox L. A. Mullick S. (2025). Characterizing HIV seroconversions among a cohort of oral PrEP users in South Africa. J. Int. AIDS Soc 28, e26421. Available online at: http://onlinelibrary.wiley.com/doi/10.1002/jia2.26421/full. 39985280 10.1002/jia2.26421 PMC11845853

[B27] Muhlbacher A. Sauleda S. Piron M. Rietz R. Permpikul P. Klinkicht M. . (2019). A multicentre evaluation of the Elecsys® HIV Duo assay. J. Clin. Virol. 112, 45–50. doi:10.1016/j.jcv.2018.11.005 30611626

[B28] Navarra A. Piselli P. Tavelli A. De Carli G. Regine V. Mammone A. . (2025). Progresses in the continuum of care of people living with HIV, using surveillance and cohort data. Italy 2012-2023. Sex Transm. Infect. 101, A1–A207. doi:10.1136/sextrans-icar-2025.4

[B29] Owen S. M. Yang C. Spira T. Ou C. Y. Pau C. P. Parekh B. S. . (2008). Alternative algorithms for human immunodeficiency virus infection diagnosis using tests that are licensed in the United States. J. Clin. Microbiol. 46, 1588–1595. doi:10.1128/JCM.02196-07 18322061 PMC2395119

[B30] Parker J. Carrasco A.-F. Chen J. (2019). BioRad BioPlex® HIV Ag-Ab assay: Incidence of false positivity in a low-prevalence population and its effects on the current HIV testing algorithm. J. Clin. Virol. 116, 1–3. doi:10.1016/j.jcv.2019.04.002 30981082 PMC6557666

[B31] Patel H. K. Duong Y.-T. Birhanu S. Metz M. Garfinkel J. Lupoli K. . (2025). Performance of national HIV testing algorithms in 14 population-based HIV impact assessment surveys: Accuracy of HIV diagnosis using a two-test strategy, with or without a tie-breaker test, in different prevalence settings 2015-2022. J. Clin. Microbiol. 63, e0117325. doi:10.1128/jcm.01173-25 41165427 PMC12710320

[B32] Patel P. Mackellar D. Simmons P. Uniyal A. Gallagher K. Bennett B. . (2010). Detecting acute human immunodeficiency virus infection using 3 different screening immunoassays and nucleic acid amplification testing for human immunodeficiency virus RNA 2006-2008. Arch. Intern. Med. 170, 66–74. doi:10.1001/archinternmed.2009.445 20065201

[B33] Pinar Bilir S. Krishnamurthy P. Karichu J. K. Blosser S. J. Hernandez D. Tchelidz T. . (2025). Estimating the economic consequences of leveraging the novel cobas HIV-1/HIV-2 qualitative nucleic acid amplification test in routine HIV testing. Clin. Inf. Dis. 81, 296–303. doi:10.1093/cid/ciaf025 39879555 PMC12448589

[B34] Pitasi M. A. Patel S. N. Wesolowski L. G. Masciotra S. Luo W. Owen S. M. . (2020). Performance of an alternative laboratory-based HIV diagnostic testing algorithm using HIV-1 RNA viral load. Sex Transm. Dis. 47, S18–S25. doi:10.1097/OLQ.0000000000001124 31895304 PMC7246040

[B35] Plebani M. (2006). Errors in clinical laboratories or errors in laboratory medicine? Clin. Chem. Lab. Med. 44, 750–759. doi:10.1515/CCLM.2006.123 16729864

[B36] Pollet D. E. Saman E. L. Peeters D. C. Warmenbol H. M. Heyndrickx L. M. Wouters C. J. . (1991). Confirmation and differentiation of antibodies to human immunodeficiency virus 1 and 2 with a strip-based assay including recombinant antigens and synthetic peptides. Clin. Chem. 37, 1700–1707. doi:10.1093/clinchem/37.10.1700 1914169

[B37] Regine V. Pugliese L. Ferri M. Santaquilani M. Suligoi B. (2025). Aggiornamento delle nuove diagnosi di infezione da HIV e dei casi di AIDS in Italia al 31 dicembre 2024. Not. Ist. Sup. Sanit. 38, 3–59.

[B38] Robert Koch-Institut (2022). “ HIV-infektion/AIDS: RKI-ratgeber,” in Epidemiologisches bulletin, vol. 24. , 3–20. Available online at: https://www.rki.de/ratgeber_hiv_aids (Accessed November 26, 2025).

[B39] Salmona M. Delarue S. Delaugerre C. Simon F. Maylina S. (2014). Clinical evaluation of BioPlex 2200 HIV Ag-Ab, an automated screening method providing discrete detection of HIV-1 p24 antigen, HIV-1 antibody, and HIV-2 antibody. J. Clin. Microbiol. 52, 103–107. doi:10.1128/JCM.02460-13 24153130 PMC3911434

[B40] Sociedad Espanola de Enfermedades Infecciosas y Microbiologia Clinica (2014). Diagnóstico microbiológico de la infección por el VIH. Available online at: https://seimc.org/wp-content/uploads/2025/06/seimc-procedimientomicrobiologia6b.pdf (Accessed November 26, 2025).

[B41] Swiss Federal Office of Public Health (FOPH), Communicable Diseases Division (2025). HIV testing guidelines 2025. Version 3, 03.09.2025.

[B42] Thorpe R. Brasher M. D. R. Bird V. R. Garrett A. J. Jacobs J. P. Minor P. D. . (1987). An improved immunoblotting procedure for the detection of antibodies against HIV. J. Virol. Methods 16, 87–106. doi:10.1016/0166-0934(87)90034-6 3301880

[B43] Tobler L. H. Kaufman E. Gefter N. Schable C. Busch M. P. (1997). Use of human immunodeficiency virus (HIV) type 1 and 2 recombinant strip immunoblot assay to resolve enzyme immunoassay anti-HIV-2-repeatably reactive samples after anti-HIV-1/2 combination enzyme immunoassay screening. Transfusion. 37, 921–925. doi:10.1046/j.1537-2995.1997.37997454018.x 9308638

[B44] UNAIDS . (2023). “ UNAIDS global AIDS update, (2023),” in Geneva: joint united nations programme on HIV/AIDS. Available online at: https://thepath.unaids.org/wpcontent/themes/unaids2023/assets/files/2023_report.pdf (Accessed November 26, 2025).

[B45] U.S. Food & Drug Administration (2023). Recommendations for evaluating donor eligibility using individual risk-based questions to reduce the risk of human immunodeficiency virus transmission by blood and blood products. Guidance for industry. Available online at: https://www.fda.gov/regulatory-information/search-fda-guidance-documents/recommendations-evaluating-donor-eligibility-using-individual-risk-based-questions-reduce-risk-human (Accessed November 26, 2025).

[B46] White D. A. E. Giordano T. P. Pasalar S. Jacobson K. R. Glick N. R. Sha B. E. . (2018). Acute HIV discovered during routine HIV screening with HIV antigen-antibody combination tests in 9 US emergency departments. Ann. Emerg. Med. 72, 29–40.e2. Available online at: https://www.ncbi.nlm.nih.gov/29310870 . 29310870 10.1016/j.annemergmed.2017.11.027

[B47] World Health Organization (2021). Consolidated guidelines on HIV prevention, testing, treatment, service delivery and monitoring: Recommendations for a public health approach; Licence: CC BY-NC-SA 3.0 IGO; World Health Organization: Geneva, Switzerland 2021. 34370423

[B48] Zucker J. Carnevale C. Rai A. J. Gordon P. Sobieszczyk M. E. (2018). Positive or not, that is the question: HIV testing for individuals on pre-exposure prophylaxis. J. Acquir. Immune Defic. Syndr. 78, e11–e13. doi:10.1097/qai.0000000000001665 29481487 PMC5953799

